# Revisiting the Two-Layer Hypothesis: Coexistence of Alternative Functional Rooting Strategies in Savannas

**DOI:** 10.1371/journal.pone.0069625

**Published:** 2013-08-12

**Authors:** Ricardo M. Holdo

**Affiliations:** 1 Divison of Biological Sciences, University of Missouri, Columbia, Missouri, United States of America; Vrije Universiteit, The Netherlands

## Abstract

The two-layer hypothesis of tree-grass coexistence posits that trees and grasses differ in rooting depth, with grasses exploiting soil moisture in shallow layers while trees have exclusive access to deep water. The lack of clear differences in maximum rooting depth between these two functional groups, however, has caused this model to fall out of favor. The alternative model, the demographic bottleneck hypothesis, suggests that trees and grasses occupy overlapping rooting niches, and that stochastic events such as fires and droughts result in episodic tree mortality at various life stages, thus preventing trees from otherwise displacing grasses, at least in mesic savannas. Two potential problems with this view are: 1) we lack data on functional rooting profiles in trees and grasses, and these profiles are not necessarily reflected by differences in maximum or physical rooting depth, and 2) subtle, difficult-to-detect differences in rooting profiles between the two functional groups may be sufficient to result in coexistence in many situations. To tackle this question, I coupled a plant uptake model with a soil moisture dynamics model to explore the environmental conditions under which functional rooting profiles with equal rooting depth but different depth distributions (*i.e.*, shapes) can coexist when competing for water. I show that, as long as rainfall inputs are stochastic, coexistence based on rooting differences is viable under a wide range of conditions, even when these differences are subtle. The results also indicate that coexistence mechanisms based on rooting niche differentiation are more viable under some climatic and edaphic conditions than others. This suggests that the two-layer model is both viable and stochastic in nature, and that a full understanding of tree-grass coexistence and dynamics may require incorporating fine-scale rooting differences between these functional groups and realistic stochastic climate drivers into future models.

## Introduction

The distribution of most terrestrial biomes can be derived from climatic variables [1], but savannas challenge this model, often occurring under conditions that can theoretically support forests [2]. Why do trees fail to competitively exclude grasses in many ecosystems, and *vice versa*? Walter's two-layer model [3] proposes that differences in soil moisture use as a function of depth results in niche partitioning (and therefore coexistence) between trees and grasses [3], [4], [5], [6]. The role of this mechanism as a general explanation for tree-grass coexistence has not been comprehensively tested. Despite this, it has gradually fallen out of favor in the savanna literature [7], [8], [9], [10], [11], giving way to demographic models, which assume that trees and grasses essentially compete for the same resources, but periodic disturbances prevent trees from completely excluding grasses [8], [12].

As originally proposed by Walter, vertical resource partitioning interacted with other tree-grass trait differences (e.g., in root morphology and water use) to promote coexistence, but only in certain savanna types [6], [13]. Subsequent work somewhat simplified the niche differentiation model by focusing on rooting separation alone, and over time this vertical resource partitioning model has become a general hypothesis for explaining tree-grass coexistence [4], [6], [7], [14], [15]. The evidence for or against such vertical partitioning in savannas has been mixed [16], [17], [18], [19], [20], and this may explain why demographic explanations have become more dominant over the past decade or so [8], [12], [21]. Fire and herbivory are well-known to be strong drivers of tree cover change in many savanna ecosystems [21], [22], [23], [24], but it is still far from clear how important or general the vertical resource partitioning mechanism is for allowing coexistence and for determining tree-grass ratios in the absence of fire. In fact, global and continental-scale studies suggest that water availability is the ultimate factor determining the upper boundary of tree cover over a wide precipitation range [2], [12], [21], and that, below a certain threshold of mean annual precipitation, resource availability is the driving force behind tree-grass coexistence. Ultimately, it still has not been satisfactorily demonstrated that niche partitioning mechanisms are incapable of explaining the savanna state, even under quite mesic conditions. In other words, are aboveground drivers such as fire and herbivory necessary for tree-grass coexistence, or are they modifiers acting upon a system that is ultimately made possible by resources alone? Second, how pervasive is niche partitioning likely to be as a viable mechanism of coexistence across broad edaphic and climatic gradients?

The empirical case against the vertical resource partitioning model includes the observation that trees and grasses sometimes show substantial rooting overlap [20], [25], [26], the suggestion that water may rarely infiltrate to deeper soil layers during the growing season [27], and the fact that grasses have deep roots and therefore possibly the same access to deep water as trees [28]. Against this, it must be considered that rooting differences may be subtle but important [7], [29], that the degree of deep infiltration during the growing season may vary systematically with climate and soils [30], and that deep grass roots may play little functional significance, except as a survival mechanism during drought [31]. Grass and tree roots have very distinct morphologies; for example, grass roots have no secondary growth or central taproot, but rather are fine and adventitious, with extremely high total root length and surface area. These morphological differences translate into functional differences in terms of the relative ability of trees and grasses to extract water as a function of depth, independently of maximum rooting depth [31].

From the theoretical perspective, few models have explicitly investigated the importance of vertical rooting separation for tree-grass coexistence. It is unclear, for example, how much rooting separation is necessary to allow coexistence, and under what conditions competition is likely to be most intense. Early theoretical models based on the two-layer model suggested that trees and grasses could coexist stably under certain conditions [4], [14], but these models relied on extreme rooting separation between the two functional groups, assuming that grasses and trees have exclusive access to topsoil and subsoil moisture, respectively. This extreme assumption, unsupported by empirical evidence [28], may have contributed to the gradual loss of support for the resource partitioning hypothesis. Models that relaxed this extreme assumption failed to predict coexistence in the absence of disturbance [10], [32]. Over the past decade or so, a number of savanna models have tended to focus on the role of disturbance (primarily fire) as a requirement for the stable maintenance of the savanna state [8], [9]. Other theoretical studies that have focused primarily on hydrological mechanisms [27], [33], [34] have, on the other hand, shown convincingly that water availability alone can predict the savanna state, but these models, motivated perhaps by a lack of apparent empirical support for vertical niche differentiation [27], have side-stepped the issue of rooting separation between trees and grasses. A common feature of these models is the stochastic treatment of rainfall inputs [33], [35]. This stochasticity, in combination with a spatially-explicit representation of local vegetation interactions and ecophysiological differences or tradeoffs between trees and grasses, allows the savanna state to persist.

In this paper I present a simple model of competition among alternative water-use strategies, defined by contrasting rooting profiles (*i.e.*, patterns of root biomass allocation as a function of depth) to test the role of vertical resource partitioning as a coexistence mechanism in savannas. The model puts aside some of the mechanisms that have already been shown by theory to promote coexistence between trees and grasses, such as phenological differences, differences in maximum rooting depth [36], and lateral competition for resources [27]. I assume that variation in root shape along the vertical axis is the key trait differentiating trees from grasses (or other functional groups, or even species within a functional group), so that the model is spatially-implicit on the horizontal axis but spatially-explicit on the vertical one. I first investigate whether the model predicts coexistence of two or more rooting profiles for a single, well-studied site (Nylsvlei, South Africa). I then test the model across a broad rainfall gradient and for two very different types of soil texture, to ask the following questions: 1) can vertical resource partitioning mechanisms provide a sufficient explanation for coexistence across a wide range of conditions, and 2) are such mechanisms more likely to offer an explanation for coexistence under some climatic and edaphic conditions than others?

## Materials and Methods

### Soil moisture dynamics and plant transpiration model

I modeled the biomass and water uptake dynamics of four competing plant rooting profiles. I assumed that different rooting profiles correspond to distinct water-acquisition strategies defined by particular root shapes or depth distributions. I assume that functional root mass (the ability of roots to acquire water), which is the key variable here, can be represented by root biomass. It should be noted that physical rooting profiles (whether measured by root length, width, or mass) do not necessarily show a direct correspondence with functional profiles [37], but I make this simplifying assumption to keep the model tractable (*i.e.*, to avoid having to allocate biomass increases to roots that absorb water and those that do not). I assume throughout the paper that the term “rooting profile” corresponds to functional root activity.

To model soil moisture dynamics, I used a modified version of Rodriguez-Iturbe *et al.*'s [38] soil moisture balance equation as the starting point for a vertically-resolved ‘bucket-type’ soil moisture model. I assumed a maximum rooting depth of 70 cm and divided the soil space into eight layers, as follows: 0–5 cm, 5–10 cm, and in 10-cm increments thereafter. The soil moisture dynamics in each layer *i* are determined by an ordinary differential equation:
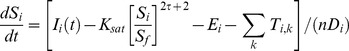
(1)where *S_i_* is the relative moisture saturation of soil layer *i*, *I_i_* is infiltration (in mm d^−1^) from the layer above (in the case of layer 1, *I* is rainfall input), *K_sat_* (in mm d^−1^) and τ are texture-dependent parameters that determine the rate of water infiltration to deeper layers [39], *E_i_* is evaporation, *T_i,k_* is transpiration (both in mm d^−1^) of rooting profile *k* from soil layer *i*, *n* is soil porosity, *D_i_* is the depth of soil layer *i* (in mm), and *S_f_* is the field capacity of the soil. The second term on the r.h.s. of eq. 1 serves as an input for the layer below, and losses from the last layer (layer 8, 60–70 cm) result in deep drainage. I assumed that evaporation only occurs in the top 5 cm, *i.e.*, layer 1 [40].

**Figure 1:**
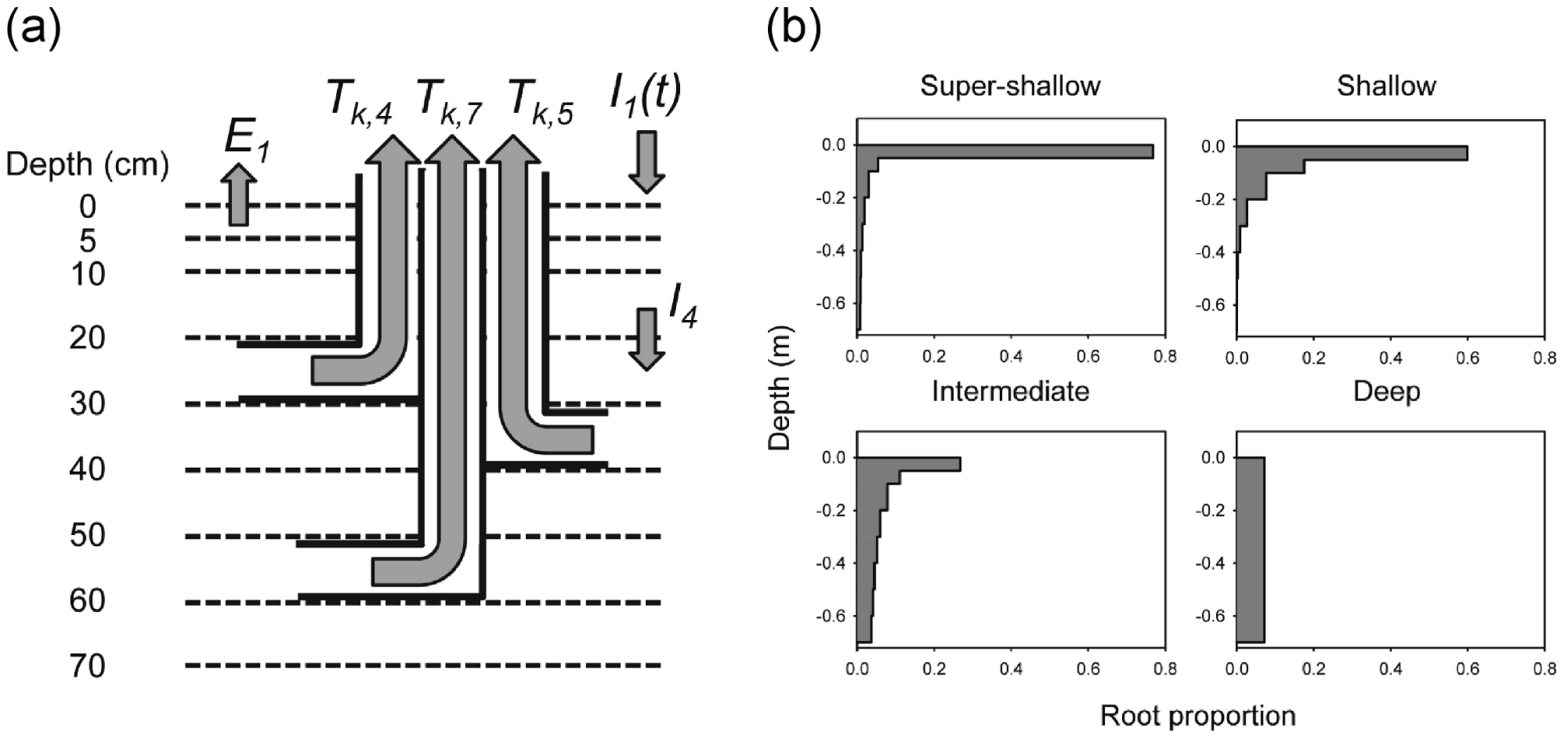
Water balance terms and rooting profiles used in the model. (a) The soil profile is partitioned into eight discrete layers for simulation of soil moisture dynamics. Transpiration is modeled according to a pipe model, with independent vessels linking each soil layer with the atmosphere. A generic functional group *k* with all root biomass allocated to soil layers 4 (20–30 cm), 5 (30–40 cm) and 7 (50–60 cm) is depicted. Water fluxes shown are rainfall inputs to layer 1 (*I_1_*(*t*)), infiltration from layer 3 to layer 4 (*I_4_*), evaporation from layer 1 (*E_1_*), and transpiration from layers 4, 5 and 7. (b) Rooting profiles for four functional groups; the maximum rooting depth is 70 cm in all cases and cumulative relative root mass equals 1.

To model plant transpiration, I assumed a pipe model in which roots allocated to a particular soil layer retain vascular independence (Fig. 1a). This pipe model framework has previously been used to inform theoretical studies of plant ecophysiology and allometry [41], [42], and is supported by empirical studies [43], [44]. The amount of water that originates from a specific soil layer and that is then transpired depends on the moisture saturation of the soil layer and the root mass allocated to it. The total transpiration for any given profile is equal to the sum of the fractions contributed by each layer. The resulting net biomass gain resulting from carbon assimilation is then distributed across the profile so as to maintain the (fixed) root shape, *i.e.*, transpiration results in biomass increases or declines but plant architecture is conserved. As a result, root biomass in a poorly-performing layer can still increase as a result of a subsidy from a layer with high uptake. I assumed that transpiration from each layer follows a Michaelis-Menton (MM) function:
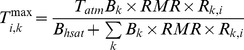
(2)Here, 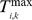 is the maximum transpiration of profile *k* from soil layer *i*, assuming that soil moisture is not limiting, *B_k_* is the total biomass (in g m^−2^) of profile *k*, *RMR* is the root mass ratio (the proportion of the total biomass that is comprised by roots, assumed equal across functional groups), *R_k,i_* is the proportional root mass allocation of profile *k* to layer *i*, and *B_hsat_* (in g m^−2^) is the half-saturation value of root biomass in the MM function. As root biomass increases, transpiration 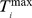 saturates at *T_atm_*, the maximum atmospheric evapotranspirational demand. This occurs as long as soil moisture *S_i_* exceeds S*, the relative soil moisture content below which stomatal conductance declines as a result of moisture stress [35], [45]. The choice of the MM was somewhat arbitrary – any saturating function might prove acceptable. The rationale for a saturating function is that the ability to transport water is limited by atmospheric demand, regardless of root conducting capacity. The nonlinear function minimizes the need to truncate transpiration rates at *T_atm_* (see below, and p. 71 in [28]). The MM function in particular is widely used in biology, both as a mechanistic and phenomenological representation of rate limitation, and has been used to model water uptake [24], [46]. Below S*, transpiration declines as follows:
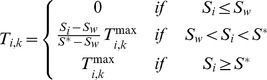
(3)This assumes that transpiration declines in a linear fashion as a function of *S_i_* between S* and *S_w_*, the wilting point [38], [45]. Below S^*^, transpiration is no longer solely under the control of atmospheric demand. The total transpiration is summed across soil layers and profiles. Because the soil layers and their respective roots are treated independently, total uptake could theoretically exceed *T_atm_* despite eq. 2. To prevent this from occurring, when 
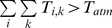
, *T_i,k_* values are rescaled proportionately so that the sum equals *T_atm_* [28]. The rescaled values are used for the transpiration term in eq. 1.

The biomass dynamics for each functional group are then given by:
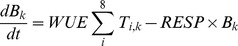
(4)Where *WUE* (in g m^−2^ per kg of water transpired) and *RESP* (in g g^−1^ m^2^) are the whole-plant water use efficiency and mass-specific respiration costs, respectively, and the *T_i,k_* values are the rescaled values.

I obtained most model parameters from published estimates (Table 1). Most of the parameters apply to soil texture and vegetation data from the Nylsvley site, an extensively-studied tropical savanna ecosystem in South Africa [28] that has often been used as a model system for savanna ecohydrology [27], [33], [34], [45]. I used published estimates of total transpiration and net primary productivity (NPP) for the site [28] to derive an aggregate value of *WUE*. I solved eq. 4 using a mean annual transpiration estimate and estimates of standing biomass obtained from [28] to obtain a value for *RESP*. I assumed that the maximum standing biomass estimates of *B* for Nylsvlei represent a steady state, and that mean respiration costs balance assimilation gains, which allowed me to set the l.h.s. in eq. 4 to 0 and solve for *RESP*. There was no parameter tuning or fitting.

**Table 1:** Parameters for the soil moisture and biomass dynamics model.

	Soil texture				
Symbol	Coarse	Fine	Units†	Description	Source(s)
*S_f_*	0.30	0.50		Field capacity	[45], [61]
*S_w_*	0.05	0.1		Wilting point	[45], [61]
S^*^	0.1	0.2		Soil moisture saturation leading to reduced transpiration	[45], [61]
*K_s_*	1100	300	mm d^−1^	Saturated conductivity	[39], [61]
τ	4.5	6.0		Exponent for conductivity function	[39], [45]
*n*	0.42	0.45		Soil porosity	
*E*	1.5	1.5	mm d^−1^	Maximum evaporative demand	[45]
*T_atm_*	3.25	3.25	mm d^−1^	Maximum atmospheric demand in mm d^−1^	[45]
*B_hsat_*	800	800	g m^−2^	Half-saturation value of *B*	Author's estimate
*RMR*	0.5	0.5		Root Mass Ratio	[28]
*WUE*	4.0	4.0	g m^−2^ kg^−1^ H_2_O	Water-use efficiency	Derived from data in [28]
*RESP*	0.001	0.001	g g^−1^ m^2^	Respiratory cost	Derived from data in [28] and eq. 4

† Empty spaces signify dimensionless parameters.

### Rainfall

To generate rainfall scenarios that are consistent with observed patterns across mean annual precipitation (MAP) gradients, I relied on North American daily climate data from Long Term Ecological Research (LTER) sites (given the lack of comparable data for African sites): Jornada (long-term MAP: 276 mm), Shortgrass (329 mm), Cedar Creek (778 mm) and Kellog (896 mm). I treated rainfall as a Poisson process, with exponentially distributed interarrival times and lognormally distributed depth. I was therefore able to summarize long-term daily rainfall across sites using three parameters: a rate parameter (λ) for the interarrival time and mean (μ) and standard deviation (σ) parameters for event depths. These parameters showed strong linear correlations with MAP (Fig. S1). This allowed me to generate time series of rainfall realizations for specific MAP values with a stochastic simulator. I assumed a mean annual rainfall of 650 mm for Nylsvlei. Although more realistic tools are available for modeling Southern African rainfall [47], the approach I used enabled me to derive rainfall sequences with reasonable event depths and interarrival times as a function of a single independent variable (MAP).

### Rooting profiles

I used a Beta distribution, rescaled to vary between 0 and 70 cm (the maximum soil depth in the model) to generate the four rooting profiles. I generated distributions in R [48] by using the *pbeta* function to obtain discretized rooting fractions in each of the eight soil layers used by the model (Fig. 1b). The distributions ranged from a ‘super-shallow’ profile (with Beta distribution parameters shape 1 = 0.1 and shape 2 = 1) where >75% of root mass was concentrated in the top 5 cm of soil, to a ‘deep’ profile (shape 1 = 1; shape 2 = 1) with a uniform distribution along the depth axis (Fig. 1b). The ‘super-shallow’ and ‘shallow’ (shape 1 = 0.5; shape 2 = 5) profiles might be thought of as a grassy/herbaceous functional group, and the ‘intermediate’ (shape 1 = 0.5; shape 2 = 1) and ‘deep’ profiles as contrasting deeper-rooted forms typical of shallow- vs. deep-rooted tree species (Fig. 1b), such as *Terminalia sericea* and *Pterocarpus angolensis*, respectively [49]. None of these profiles were fit to real rooting data, but rather represent a range of possible shapes that are qualitatively consistent with profiles reported in the literature [28], [50].

### Model testing

I solved the model equations numerically in Microsoft Visual C++ using a daily time step (the full code is available in Source Code S1). I ran the simulations to steady state in every case (100–1000 years). I initially ran simulations with a number of preliminary scenarios designed to test the robustness and soundness of the model. In these simulations, I assumed that parameters unrelated to root shape, *i.e.*, *RMR*, *WUE*, and *RESP* were identical across rooting profiles. I conducted the following simulations:

- A single 1000-year run with each rooting profile as the sole strategy, to establish that each profile was viable in isolation.
- Simulations with all four profiles competing. For each run (N = 5, 500 years), the starting biomass of each profile was chosen from a uniform random distribution (range: 1000–2000 g m^−2^).
- To test the robustness of the model to the particular uptake function chosen, I also conducted a pair of 1000-year runs, the first assuming the default MM uptake function and the second assuming a linear uptake function (i.e., with *B_hsat_* = 0) .
- To investigate the role of the stochastic nature of rainfall inputs, I conducted 100-year simulations with all functional groups present, but assumed a constant, deterministic rainfall input, equal to MAP divided by 365 to give an input of 1.78 mm d^−1^. I set initial biomass values for all rooting profiles to 1000 g m^−2^.
- I conducted a global sensitivity analysis (GSA), consisting of 400 runs (of 500 years each), in which 13 parameters were assigned offset values (multiplied by their default values from Table 1) drawn from a uniform random distribution with range 0.8–1.2 (*i.e.*, each parameter deviated by a maximum of ±20% of its default value). I included all 12 model parameters from Table 1 in the analysis, plus an additional parameter (default value = 0.5 g m^−2^ kg^−1^ H_2_O) that determined the *WUE* advantage (in relation to the *WUE* of the other three profiles) of the super-shallow profile. I chose this profile for testing the effect of varying *WUE* because it was the most likely to go extinct during simulations, and I wanted to explore the ability of *WUE* to ‘rescue’ this strategy. The primary objective of the GSA was to explore the parameter space of coexistence. I calculated the proportion of runs for which two or more profiles coexisted after 500 years, and the median number of coexisting profiles.

In all of the above simulations, I assumed equal *WUE* (set at the default value, Table 1) for all rooting profiles, and assumed the environmental conditions of the Nylsvlei site (650 mm MAP and coarse-textured soils).

#### Single site coexistence

I next ran a series of simulations in which each rooting profile was assigned a slightly higher WUE than the other three (4.5 instead the of the default value of 4.0 g m^−2^ kg^−1^ H_2_O). The competitive exclusion principle states that two species with identical traits cannot coexist stably [51]; over the long term, a slight advantage in one species (e.g., a marginally higher intrinsic population growth rate) will lead to competitive exclusion. In most of the simulations in this paper, therefore, I parted from the assumption that one of the four profiles under consideration has a slight advantage in *WUE* (*i.e.*, higher biomass growth per g of water transpired), and then tested the ability of the other profiles to persist and invade despite this *WUE* disadvantage. For each set of *WUE* conditions (out of four), I ran 500-year simulations (N = 5 runs) for the Nylsvlei environmental conditions and tested for coexistence at the end of each run. In these and other cases I treated a final biomass of <1 g m^−2^ as extinction. To test the robustness of the coexistence results, I repeated the simulations under the following conditions: the strategy with the *WUE* advantage was run alone to steady state (from an initial biomass of 4000 g m^−2^) for 100 years, and the remaining strategies were then introduced to the system with small initial biomasses (10 g m^−2^) so as to test their ability to invade the established profiles (*i.e.*, increase) over 100 additional years. For completeness (and to further test the model), I also ran simulations in which the established profile tried to invade itself, but with a lower (default) value of *WUE*.

#### Coexistence across rainfall and edaphic gradients

To test the robustness of the coexistence results across a range of rainfall and soil conditions, I ran the model (500-year simulations, N = 5 runs, initial biomass drawn randomly from the interval 1000–2000 g m^−2^) across a simulated rainfall gradient spanning 300–1500 mm y^−1^ (in intervals of 300 mm y^−1^) under two soil texture scenarios: a coarse-textured soil substrate (based on Nylsvlei) consisting of sand/loamy sand soils, and a fine-textured substrate consisting of a higher clay fraction. These simulations build on the GSA, with the difference that this exercise varied external environmental conditions rather than intrinsic biological parameters. Variation in rainfull inputs was achieved purely by varying MAP in the rainfall generator; simulating contrasting soil substrates required adjusting some of the model parameters (Table 1).

## Results

### Model testing

**Figure 2:**
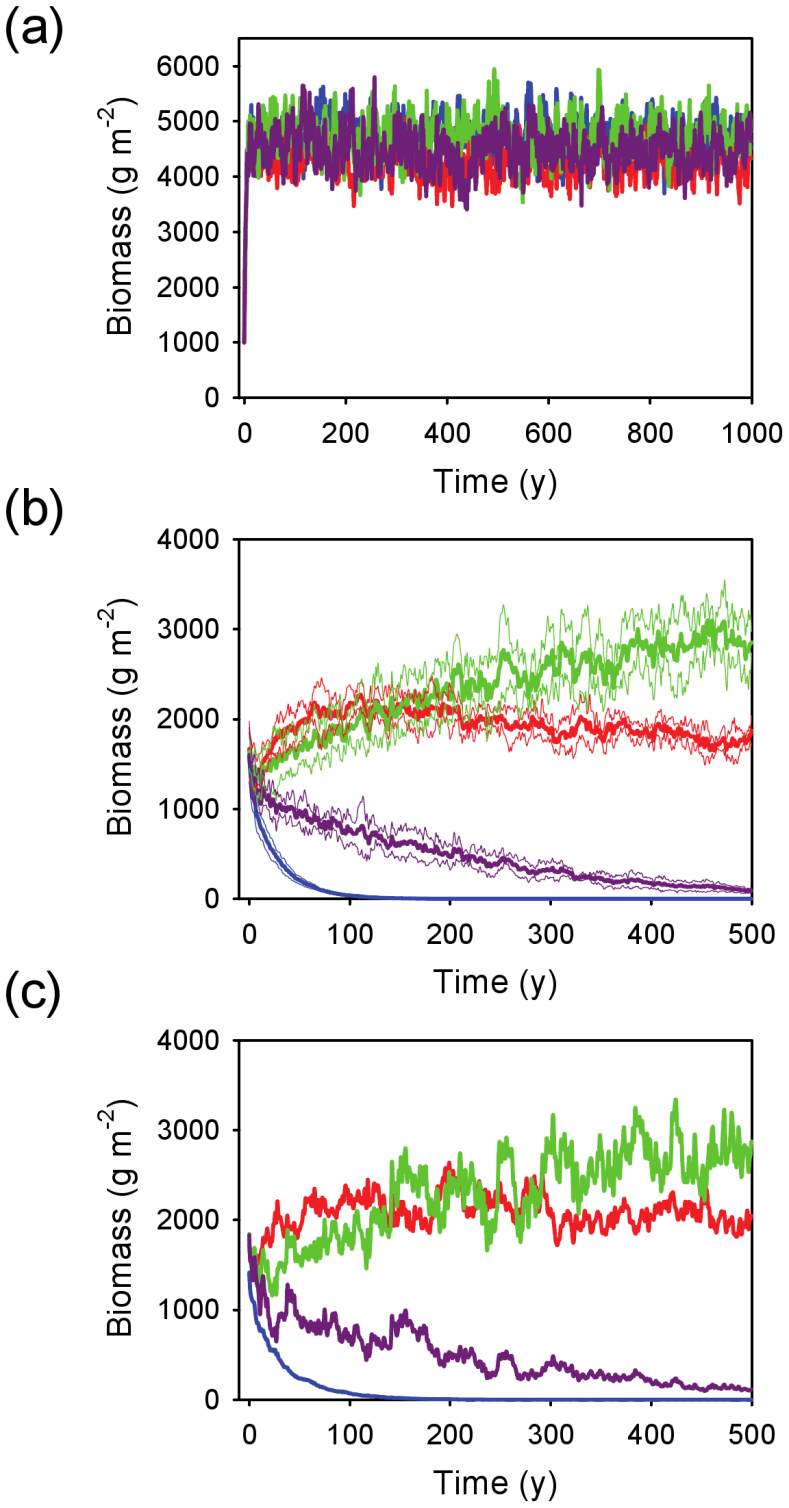
Time series of biomass dynamics for four rooting profiles assuming coarse-textured soils and MAP = 650 mm y^−1^. Model outcomes with (a) each profile in isolation (note: profiles are plotted together for comparision); (b) all profiles initially present, with equal *WUE*; (c) the same scenario as b, but assuming a linear uptake function instead of the default Michaelis-Menton function. Thick lines show mean values and thin lines (where present) show the mean ± 1 SD across runs. Profile key: blue = super-shallow, red = shallow, green = intermediate, purple = deep.

**Figure 3:**
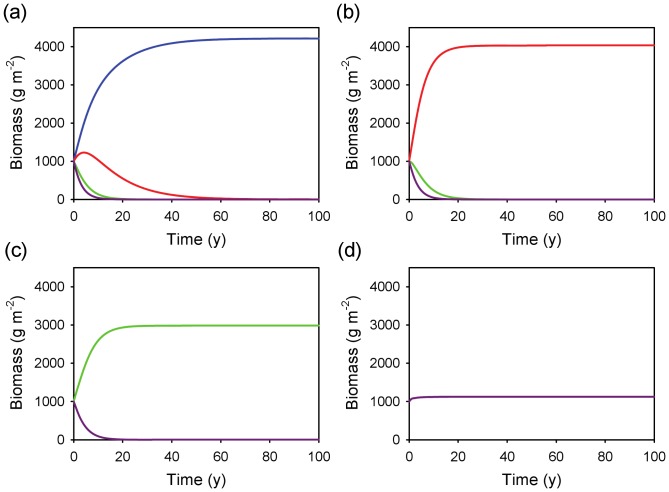
Time series of biomass dynamics for four rooting profiles on coarse-textured soil under a deterministic (constant) rainfall regime. Model outcomes (a) with all profiles included; (b) after removal of the winning profile (*i.e.*, super-shallow) from a; (c) after removal of the winning profile (*i.e.*, shallow) from b; (d) with the deep profile in isolation. MAP was fixed at  = 650 mm y^−1^ and divided by 365 to generate a constant daily rainfall input. Profile key: blue = super-shallow, red = shallow, green = intermediate, purple = deep.

All rooting profiles were viable when run in isolation under the conditions present at Nylsvlei (Fig. 2a), rapidly converging on a standing biomass of around 4500 g m^−2^ in all cases. This represents total (above plus belowground) biomass, and given the assumed *RMR* of 0.5 represents about 2250 g m^−2^ of aboveground biomass. This value is compatible with the estimate of tree standing biomass of 1627 g m^−2^ (which excludes grasses, so is an under-estimate of total aboveground biomass) reported by Scholes and Walker [28] for this site. When all profiles were included, the shallow and intermediate profiles achieved steady-state (nonzero) biomass values, with the super-shallow and the deep profiles going extinct rapidly and gradually, respectively (Fig. 2b). The runs with linear uptake did not differ qualitatively from those with MM uptake, suggesting that the model is robust to the particular uptake function chosen (Fig. 2c). In the deterministic rainfall scenario, the super-shallow rooting profile excluded all others within 100 years (Fig. 3a). I repeated the simulation by excluding the winning strategy after each run. In every case, a single profile (always the shallowest one) excluded all others (Fig. 3b–d), suggesting that the stochastic nature of the rainfall inputs is a key ingredient for the coexistence of alternative profiles.

In the sensitivity analysis, coexistence of two or more strategies (median = 2) persisted after 500 years in 56% of the 400 runs. The outcome of the GSA was strongly influenced by the value of a single parameter, however. Variation in *S_f_* (field capacity) accounted for 70% of the variance in total biomass after 500 years. Given that field capacity is both a readily-measurable biophysical parameter and one that is closely correlated (*i.e.*, not independent of) with other ecohydrological parameters such as the wilting point (*S_w_*) and saturated conductivity (*K_s_*), it was clear that the GSA was too conservative (in other words, “generous” in its exploration of parameter space) in its assumption of independent parameter variation. To restrict this dominant effect of *S_f_*, I examined a subset of results in which *S_f_* was restricted to values that fell within 5% of its default value of 0.30 (104 of the 300 runs). For this subset of the GSA runs, coexistence of two or more strategies (median = 3) persisted in 96% of the runs, suggesting that the basic coexistence result is robust.

### Single site coexistence

**Figure 4:**
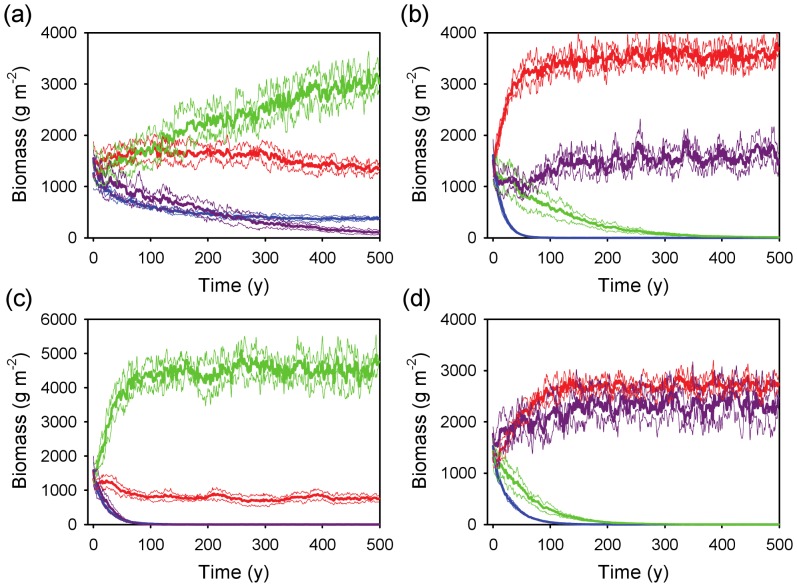
Time series of biomass dynamics for four rooting profiles assuming coarse-textured soils and MAP = 650 mm y^−1^. Each panel assumes a water use efficiency advantage (*WUE* = 4.5 for the target profile vs. 4.0 g m^−2^ kg^−1^ for the other three) for a different target profile: (a) super-shallow, (b) shallow, (c) intermediate, and (d) deep. Thick and thin lines represent the mean ± 1 SD (N = 5 runs) of total (above and belowground) biomass. Profile key: blue = super-shallow, red = shallow, green = intermediate, purple = deep.

**Figure 5:**
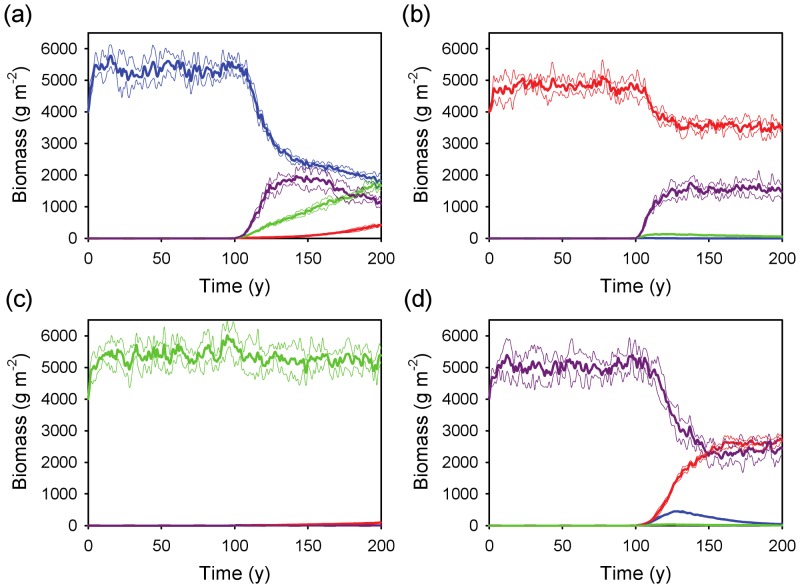
Invasibility of each rooting profile at steady state. Model outcomes are shown for (a) super-shallow, (b) shallow, (c) intermediate, and (d) deep profiles. Simulations are based on 100 years with no competition, followed by an introduction of 10 g m^−2^ of each of the other three profiles. Each profile being invaded has a *WUE* advantage over the others that matches what is shown in panels a–d of Fig. 4. Thick and thin lines represent the mean ± 1 SD (N = 5 runs) of total (above and belowground) biomass. Profile key: blue = super-shallow, red = shallow, green = intermediate, purple = deep.

Two or more rooting profiles coexisted under each of the four *WUE* conditions (Fig. 4). When the super-shallow profile had a slightly higher *WUE* than the other profiles, all profiles were still present after 500 years (although the deep profile was still declining; Fig. 4a). When the shallow profile had higher *WUE* than the others, it coexisted with the deep profile (but not the other two; Fig. 4b). A higher *WUE* for the intermediate profile allowed coexistence with the shallow one (Fig. 4c), and a higher *WUE* for the deep profile allowed it to coexist with the shallow profile, but not the other two (Fig. 4d). Two general results were that: i) the super-shallow profile was not viable unless it had a *WUE* advantage; and ii) profiles that were dissimilar (shallow and deep) were more likely to coexist than those that were more similar (shallow and intermediate or intermediate and deep), as predicted by the competitive exclusion principle. The coexistence results were robust, as suggested by the ability of alternative profiles to invade the dominant one when the latter was at steady state (Fig. 5).

#### Coexistence across rainfall and edaphic gradients

**Figure 6:**
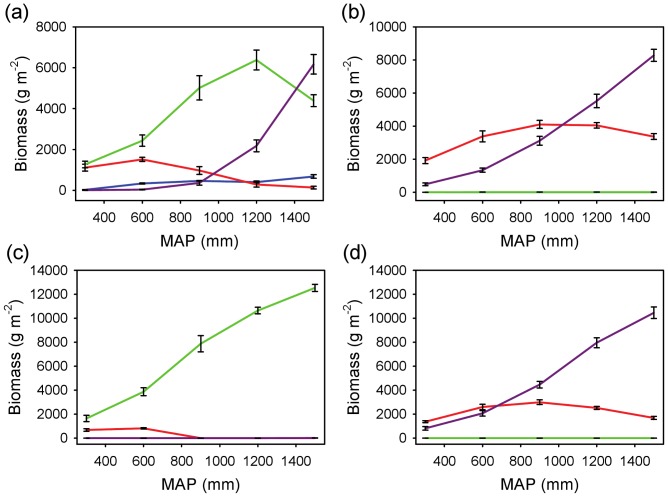
Steady-state biomass for the four rooting profiles as a function of MAP on coarse-textured soil. Each panel assumes a water use efficiency advantage (*WUE* = 4.5 for the target profile vs. 4.0 g m^−2^ kg^−1^ for the other three) for a different target profile: (a) super-shallow, (b) shallow, (c) intermediate, and (d) deep. The steady-state values (mean ± SD) are based on mean total (above and belowground) biomass across five runs. Profile key: blue = super-shallow, red = shallow, green = intermediate, purple = deep.

**Figure 7:**
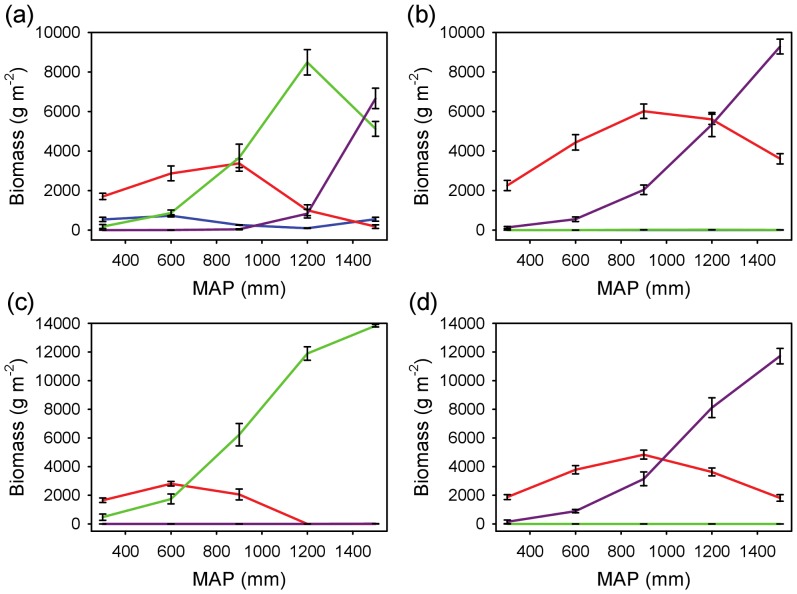
Steady-state biomass for four rooting profiles as a function of MAP on fine-textured soil. Each panel assumes a water use efficiency advantage (*WUE* = 4.5 for the target profile vs. 4.0 g m^−2^ kg^−1^ for the other three) for a different target profile: (a) super-shallow, (b) shallow, (c) intermediate, and (d) deep. The steady-state values (mean ± SD) are based on mean total (above and belowground) biomass across five runs. Profile key: blue = super-shallow, red = shallow, green = intermediate, purple = deep.

The coexistence results were also robust to variation in rainfall and soils (Figs. 6 and 7). Two or more rooting profiles (usually the shallow and deep ones) were able to coexist (depending on which of the four was assumed to have a *WUE* advantage) across a broad precipitation gradient on both coarse-textured (Fig. 6) and fine-textured (Fig. 7) soils. Overall, the combined biomass of all profiles (those that remained viable) increased as a function of MAP (Figs. 6 and 7), and increases in MAP led to sequential switches in dominance from shallower to deeper profiles. Therefore, as profile depth increases (shallow to intermediate to deep), the model predicted that the conditions that tend to favor it tend to shift to the moister end of the MAP gradient. The model results suggest that each profile has an optimum set of conditions that will allow it to dominate. In Fig. 6a, the shallow profile has a biomass peak at about 600 mm y^−1^, whereas the intermediate profile peaks at about 1200 mm y^−1^. The deep profile continues to increase in peak biomass beyond this range (Fig. 6). The location of the peaks appears to depend on the relative *WUE* values of the different groups, and to a lesser extent, on the soil substrate (Figs. 6 and 7). The simulations also suggested that the fine-textured soil conditions are relatively more favorable to shallower rooting profiles than to deeper ones (Fig. 7), as expected by the slower rate of infiltration in fine-textured soils.

## Discussion

The model results suggest that differences in plant rooting profiles are sufficient to explain coexistence when water is the limiting resource under realistic conditions, without the need to invoke the ‘exclusive-use’ premise [3], [14]. It goes further, moreover, by suggesting that multiple rooting strategies may coexist when a single resource is limiting for extended periods of time, even when rooting profiles show quite subtle differences in allocation as a function of depth (compare the shallow and intermediate strategies in Fig. 1b). The two-layer model, as previously interpreted, largely relied on the notion of ‘exclusive access’ to subsoil water on the part of trees, and the superior performance of grasses in topsoil layers, at least in theoretical models [3], [14]. Though these characteristics may well apply in many situations—in fact, we do know that grasses are able to outcompete trees in upper soil layers [31]—, their absence does not necessarily preclude coexistence. Beyond the question of tree-grass coexistence, the results have implications for interactions among tree species that coexist locally, given the diversity in root shape and allocation as a function of depth encountered in this growth form [15], [49]. The results suggest that the original premise of the Walter two-layer model, *i.e.*, that niche differentiation can explain coexistence, is valid. Coexistence is possible even with substantial rooting overlap and quite strong competitive interactions, provided that the shapes of the functional rooting profiles differ sufficiently. The underlying mechanism proposed here is not new: it is well known that spatial and temporal heterogeneity in resource supply can promote species coexistence [52]. Plant species and functional groups exhibit clear differences in depth-specific rooting profiles [49], [53], and substantial spatiotemporal heterogeneity in soil moisture availability occurs in real systems. The relative competitive advantage gained by allocating roots to alternative depths therefore shifts over time, resulting in a dynamic equilibrium for competing strategies.

Previous efforts to model tree-grass dynamics by accounting for rooting differences alone have often failed to predict coexistence [10], [32]. A key requirement for coexistence is the occurrence of deep infiltration events. As demonstrated here, the stochastic nature of precipitation inputs (which lead to these events) is critical for coexistence, but a reasonably realistic representation of the temporal dynamics of the rainfall function may also be critical [33], [38]. Jeltsch *et al.* [32] used both deterministic and stochastic rainfall functions in a tree-grass dynamics model, but found that stochasticity alone was insufficient to generate coexistence. A better characterization of interarrival times and storm depths allows for infrequent but intense rainfall events (which result in deep recharge, and may therefore be critical for the success of deep-rooted strategies), unlike stochastic functions that simply ‘spread out’ the supply of water over time. When large precipitation events occur on sandy soils, significant infiltration can occur, opening up deeper soil layers as viable sources of moisture. Walter understood that, under certain conditions, the deeper roots of trees would allow them to capture this excess moisture, particularly under more mesic conditions, therefore allowing them to coexist with grasses despite the fact that grasses have higher transpiration rates than trees and are therefore the superior competitor of the two growth forms [5], [6]. In addition to the timing and size of precipitation events, deep infiltration is a function of plant uptake, evaporative demand, and edaphic factors such as soil texture and pore size [30], [38]. Rooting separation may therefore be the result of opportunities opening up for the exploitation of a new resource (*i.e.*, subsoil water) rather than a consequence of competition for a limiting resource.

Most authors will concede that niche-based explanations for the savanna state may well play an important role under certain conditions [7], [11], [27], [28]. Many stable isotope-based studies have in fact shown clear differences in functional rooting profiles between trees and grasses and among functional groups, such as C_3_ forbs and C_4_ grasses [16], [17], [18], [54], [55], although it has been suggested that such partitioning tends to occur in systems where precipitation falls in the non-growing season, which is not the case in (for example) the extensive sub-Saharan African savannas [7]. The more limited amount of work conducted in African savannas does not appear to support the two-layer model [19], [20], [25], but upon closer scrutiny it appears that the approaches available may usually only be adequate for quantifying coarse differences in rooting profiles (a consequence of the difficulty of studying belowground patterns and processes). More painstaking studies have shown that trees and grasses may exhibit subtle rooting differences as a function of depth [29], [50], and that tree and grass profiles do differ, even when there is substantial overlap [19], [28]. These small differences may be all that is required to allow long-term coexistence.

The fact that coexistence occurs across a wide environmental range does not necessarily mean that rooting separation is the primary factor responsible for the savanna state under all or even most conditions. The model results suggest that the viability of alternative rooting strategies (as measured by the functional diversity in Fig. 4c,d) varies as a function of rainfall and soil texture. The fact that the various rooting profiles exhibit biomass optima as a function of MAP suggests that any two strategies will tend to have equal biomass where their unimodal curves intersect. At this point, which will tend to occur in areas of intermediate MAP rather than at the extremes (Figs. 6 and 7), functional “diversity” is maximized. This suggests that niche partitioning generated by rooting differences may be a more plausible strategy under intermediate (*i.e.*, neither too dry nor wet) conditions. At the dry and wet ends of the spectrum, shallow-rooted and deep-rooted strategies are favored, respectively, as has been previously noted [36]. In arid and semi-arid savannas, this may contribute to explain the upper bound of tree cover that is associated with MAP [12], [21]. Trees in semi-arid environments may have to ‘choose’ between two poor options: i) one in which they maintain a sub-optimal deep-rooted strategy (e.g., imposed by structural or architectural constraints), or ii) if rooting allocation is plastic, one in which they exploit shallow soil layers and compete directly with grasses. Either case is likely to lead to reduced tree biomass. The reality probably lies somewhere between these two extremes: trees have been shown to be opportunistic and malleable in their ability to shift their functional rooting profile [50], [55], but this ability has limits: the Serengeti plains, for example, support grasses but are too shallow to support trees [56]. In either case, trees and grasses are competing for a limiting resource: in the first case exploitative competition in topsoil leads to lower infiltration, and in the second case interference competition occurs within the soil layers where they overlap spatially. As MAP increases, spatial segregation of the resource increases, leading to greater opportunities for differentiation. At the upper end of the MAP boundary, high infiltration rates favor trees over grasses, and the savanna state is more likely to begin to give way to woodland or forest. At this point, other mechanisms are likely to begin to play a greater role, such as competition for light [9] and fire [12], [21], [24]. Over time, increasing tree and declining grass biomass in mesic sites may lead to fire suppression and species turnover from low-LAI (leaf area index) savanna species to high-LAI forest species, with light limitation eventually leading to a conversion to forest [23]. Although I do not include these mechanisms in this simple model, I propose that below-ground niche differentiation may continue to be an important component of savanna stability and species coexistence under quite mesic conditions.

### Other considerations

As outlined above, the results of the model presented here (in combination with prior work [32]) suggest that the outcome of competitive interactions are influenced by the precise nature of the stochastic function describing precipitation events. Precipitation events can be described in terms of their timing and size [15], but also by the relationship between these two variables, given that times between storms may be correlated with the size of a preceding precipitation event. Alternative stochastic functions (including more realistic, site-specific rainfall generating functions, e.g., [47]) to the one used here and elsewhere need to be tested to explore the sensitivity of coexistence outcomes to variation in the timing and size of precipitation events. This may be particularly important at the dry end of the MAP spectrum, where plants can exhibit threshold responses [15] to precipitation pulses (and therefore water uptake becomes increasingly sensitive to the frequency distribution of event sizes).

For simplicity, I have omitted other key variables from this version of the model, most notably seasonality and plant phenology. Different function groups and species exhibit alternative responses to the onset of the dry season in savanna systems, trees often flushing before grasses and retaining their leaves later into the dry season, which allows them to opportunistically respond to late precipitation events [28], [57]. Some savanna tree species are more drought-deciduous than others [28]. Niche partitioning along the temporal axis has been proposed as an alternative to the two-layer model [7], [11]. I have ignored this seasonal axis here, but it should be incorporated into a more comprehensive model of tree-grass water use and partitioning, given that the vertical distribution of soil moisture (and therefore the relative advantage of shallow and deep-rooted species) varies seasonally. There are other elements I have left out in this model that have the potential to shift its quantitative conclusions. I ignore the role of stem flow for subsoil water recharge and changes in infiltration due to soil “capping” as the proportion of bare soil increases [4], the role of tree canopies on microclimate and evaporative demand in the grass layer [14], changes in root mass ratios along climate gradients [58], hydraulic redistribution by trees [59], changes in evaporative demand correlated with MAP, and plasticity in functional rooting profiles, particularly in trees [15], [50]. I deliberately left out these factors to test the hypothesis that rooting separation can be a dominant explanation for the savanna state, although I note that the exploration of rooting niche space across environmental gradients requires further analysis.

### Conclusions and broader implications

A simple vertically-explicit model suggests that the relative importance of tree-grass competition may vary systematically along environmental gradients, and this may be an important insight for developing a predictive understanding of tree-grass dynamics under a wide range of conditions. I suggest that explicitly incorporating a mechanistic representation of tree and grass vertical rooting profiles and modeling soil moisture dynamics as a function of depth may be important for improving predictive models of savanna dynamics across a wide range of edaphic and precipitation conditions, including novel climate regimes. Hydrologic templates are shifting rapidly as a result of climate change-induced shifts in the amount and distribution of rainfall inputs and evapotranspirational demand [60]. If there are indeed strong and consistent differences in rooting profiles among functional groups and species, who will be the winners and losers of such changes at the community level? An important next step is to move beyond the debate about tree-grass coexistence and work towards the development of synthetic models that incorporate all the relevant factors necessary to move towards useful predictive model of tree-grass dynamics [7]. I propose that a systematic analysis of rooting profiles along key environmental gradients may be an important aspect of this process.

## Supporting Information

Figure S1 **Coefficients of stochastic rainfall generator as a function of mean annual precipitation (MAP) across four North American LTER sites.** (a) rate λ of the exponential distribution describing interarrival tS1imes between precipitation events, (b) mean μ and (c) standard deviation σ of the lognormal distribution describing event size. Key to LTER sites: J = Jornada, S = Shortgrass, CC = Cedar Creek, and K = Kellog.
(DOCX)

Source Code S1 **Source code.**
(ZIP)
