# Supplementary material for: Revisiting the Two-Layer Hypothesis: Coexistence of Alternative Functional Rooting Strategies in Savannas
Source: PLoS One. 2013 Aug 12;8(8):e69625. doi: 10.1371/journal.pone.0069625 (PMC3741314; doi:10.1371/journal.pone.0069625)
Supplement: Figure S1 — Coefficients of stochastic rainfall generator as a function of mean annual precipitation (MAP) across four North American LTER sites. (a) rate λ of the exponential distribution describing interarrival tS1imes between precipitation events, (b) mean μ and (c) standard deviation σ of the lognormal distribution describing event size. Key to LTER sites: J = Jornada, S = Shortgrass, CC = Cedar Creek, and K = Kellog. (DOCX) [file pone.0069625.s001.docx]

**Fig. S1.** Coefficients of stochastic rainfall generator as a function of mean annual precipitation (MAP) across four North American LTER sites: (a) rate λ of the exponential distribution describing interarrival tS1imes between precipitation events, (b) mean μ and (c) standard deviation σ of the lognormal distribution describing event size. Key to LTER sites: J=Jornada, S=Shortgrass, CC=Cedar Creek, and K=Kellog.
